# Ileosigmoid knot: a case report and literature review of 280 cases

**DOI:** 10.4103/0256-4947.55173

**Published:** 2009

**Authors:** Norman O. Machado

**Affiliations:** From the Department of Surgery, Sultan Qaboos University Hospital, Muscat, Oman

## Abstract

Ileosigmoid knotting, also known as compound volvulus or double volvulus, is a rare cause of intestinal obstruction. In this condition the ileum wraps around the base of the sigmoid colon and forms a knot. Ileosigmoid knotting is an unusual entity in the West, but is comparatively common in certain African, Asian and Middle Eastern nations. The condition is serious, generally progressing rapidly to gangrene. Awareness of the condition is essential for prompt diagnosis and optimal management. This report describes a case in a 60-year-old male and describes the management of this rare condition. An additional 280 recent cases in the English literature are reviewed as to etiopathogenesis, presentation, diagnostic modalities, surgical interventions and outcome.

Ileosigmoid knotting (ISK) is a rare cause of intestinal obstruction that rapidly progresses to gangrene of the ileum as well as the sigmoid colon.^1^ Preoperative diagnosis is difficult because of its infrequency and atypical radiographic findings.^2^,^3^ It is essential to differentiate it from sigmoid volvulus because endoscopic reduction is a contraindication in ISK. In recent years, CT has been helpful in making a preoperative diagnosis. Generalized peritonitis and sepsis is the main cause of poor outcome.^3^,^4^ After hemodynamic stabilization, immediate surgical intervention is the need of the hour.

## CASE

**Figure 1:**
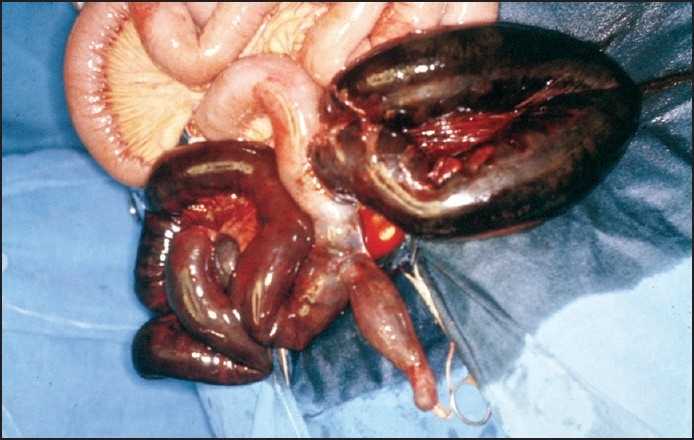
Ileosigmoid knot with gangrenous ileum and sigmoid colon and Meckel's diverticulum.

A 60-year-old male patient with a history of chronic constipation was admitted with severe lower abdominal pain of 24-hour duration. This was associated with progressive abdominal distension and vomiting. He was in distress and was dehydrated. Examination revealed a BP of 100/60 mm Hg, pulse rate of 98/min, and temperature of 38.5° C. Abdominal examination revealed diffuse abdominal distension, tenderness, and guarding. Laboratory data showed a white cell count of 20000/mm^3^, hemoglobin level of 9.6 gm%, C-reactive protein of 12.7 mg/dL (normal range, <0.3 mg/dL). Plain film of the abdomen showed dilatation of the small intestine with a gas and air fluid level, with an associated moderately distended, and obstructed sigmoid loop. A CT scan was planned, but could not be carried out for technical reasons. The patient was resuscitated with IV fluids and started on IV cefuroxime and metronidazole and was taken up for laparotomy 4 hours after admission for suspected ISK based on both the small gut and sigmoid colon obstructive features on plain x-ray of the abdomen. On laparotomy, hemorrhagic fluid was encountered and the ileum and sigmoid colon was found to be gangrenous with a 360° clockwise twist of the ileum around the sigmoid colon. In addition, a Meckel diverticulum with a small band was found at the base of the twist (Figure 1). Following a 100-cm resection of gangrenous ileum and sigmoid colon, a primary anastomosis of the small gut and colon was carried out. Except for a minor wound infection patient made an uneventful recovery and was discharged after 10 days.

## DISCUSSION

Parker is credited with having described the first patient with ISK in 1845.^1^ The first patient from the Asian subcontinent was reported by Paul in 1940, an account of which was given by Shepherd.^2^ Since then over 280 cases have been reported in the English literature.^2^–^9^ Three factors are responsible for the ileosigmoid knot: a long small bowel mesentery and freely mobile small bowel; a long sigmoid colon on a narrow pedicle; and finally the ingestion of a high bulk diet in the presence of an empty small bowel.^2^–^8^ When a semi-liquid bulky meal progresses into the proximal jejunum it increases the mobility of the intestine and the heavier segments of the proximal jejunum fall into the left lower quadrant. The empty loops of ileum and distal jejunum twist in a clockwise rotation around the base of a narrow sigmoid colon. Further peristalsis forms an ileosigmoid knot with two closed loop obstruction, one in the small bowel and other in the sigmoid colon.^2^–^6^ Evidence for the mechanism is suggested by studies carried out on Bagandans in Uganda who eat once a day and Muslims who eat a single daily meal during the Ramzan fast.^2^,^4^ ISK is predominately seen in males (80.2%) with a mean age of 40 years (range, 4-90 years) (Table 1). Besides the above anatomic prerequisites, the literature reveals the evidence of other secondary causative factors including late pregnancy, transmesenteric herniation, Meckel diverticulitis with a band, ileocecal intussusceptions, and floating cecum (Table 2).^3^,^4^,^7^,^8^ Among the 280 patients reviewed, 4.3% were children under the age of 16 years.^9^–^11^ While ISK is predominately reported in certain African, Asian, and Middle-East nations, it is a rare occurrence in the white population.^10^

**Table 1:** Demographics and clinical features.

Author (year)	No. of cases	Age mean/range	Sex M:F	Duration of symptoms in hours mean/range	Symptoms						Pre-diagnosis
					P	D	V	C	M	S	
Bawa, 2008	1	26	1:0	>240	1	1	1	1	-	-	0
Atamanalp, 2006	9	10.6 7-16	7:2	24-72 45.3	9	9	9	9	3	3	0
Berrebi, 2006	1	9	0:1	12	1	1	1	-	-	1	0
Hirano, 2005	1	75	1:0	28	1	1	1	-	-	-	1
Atamanalp, 2004	63	47 7-75	47:16	12-120 46.6	63	60	53	63	9	38	0
Hashimato, 2004	2	32 4-60	2:0	26	1	2	-	-	1	-	1
Tamura, 2004	1	78	1:0	?	1	1	-	-	-	-	1
Raveenthiran, 2001	7	43 30-60	6:1	6-240 (53)	7	6	5	7	-	4	5
Akgun, 1997	16	45 20-90	11:5	1-144 (100)	16	15	14	-	-	9	?
Alver, 1993	68	49 18-79	57:11	24-144 (33)	68	64	59	64	-	38	0
Miller, 1992	1	41	0:1	2	1	0	1	-	-	0	0
Puthu, 1991	7	40	4:3	8-72 (36)	7	7	7	-	-	2	2
Kakar, 1981	11	38	10:1	?	11	3	11	?	?	4	2
Shephard, 1967	92	42 11-75	78:14	6-76 (18)	92	90	?	?	?	?	?

P-Pain; D-Distension; V-Vomiting; C-Constipation; M-Malaena; S-Shock

**Table 2:** Secondary predisposing factors and investigations.

Author	No.	Predisposing factors LP	PS/Ad	MD	FC	IH	NS	Investigations X-ray	CT
Bawa, 2008	1	-	-	-	-	-	1	1	0
Atamanalp, 2006	9	-	-	-	-	-	9	9	0
Berrebi, 2006	1	-	-	-	-	-	1	1	1
Hirano, 2005	1	-	-	-	-	-	1	1	1
Atamanalp, 2004	63	2	10	-	1	-	-	41	0
Hashimato et al, 2004	2	-	-	-	-	-	2	2	2
Tamura et al, 2004	1	-	1	-	-	-	-	1	1
Raveenthiran, 2001	7	-	-	-	-	-	7	7	-
Akgun, 1997	16	-	-	-	-	-	16	16	-
Alver, 1993	68	4	12	1	1	4	-	56	-
Miller, 1992	1	-	1	-	-	-	-	1	-
Puthu, 1991	7	-	-	1	-	-	-	7	-
Kakar, 1981	11	-	-	-	-	-	-	8	-
Shephard, 1967	92	-	-	-	-	-	92	NS	-

LP-late pregnancy; PS-previous surgery; Ad-adhesions; MD-Meckel's diverticulum; FC-floating caecum; IH-internal herniation; NS-not specified; CT-CT scan

ISK has been categorized into three types. In type I, the ileum (active component) wraps itself around the sigmoid colon (passive component) in a clockwise or anticlockwise direction (type A when clockwise and type B when anticlockwise). In type II, the sigmoid colon (active component) wraps itself around a loop of ileum (passive component) in a clockwise or anticlockwise direction. In type III, the ileocecal segment (active component) wraps itself around the sigmoid colon (passive component).^2^,^4^ The most common type of ISK reported is type I (53.9% to 57.5%), followed by type II (18.9% to 20.6%), type III (1.5%), and others undetermined. The direction of torsion is clockwise in 60.9% to 63.2% of cases.^4^,^8^,^9^ The torsion is 360° in 52.9%, 360°×2 in 19.1%, and 360°×3 in 5.9%.^4^,^8^

ISK can rapidly progress to gangrene of the ileum as well as of the sigmoid colon.^2^,^3^,^4^ Generalized peritonitis, sepsis, and dehydration are the principal complications. The predominant symptoms and signs of presentation include abdominal pain and tenderness (100%), abdominal distension (94% to 100%), nausea and vomiting (87% to 100%), rebound tenderness (69%),^4^ and shock (0% to 60%), where it was specified (Table 1).

Despite the critical condition, preoperative diagnosis is not easy. While in recent years a preoperative diagnosis has been made more often, it was a rarity in the past in most cases (0% to 28%). The diagnostic difficulty is partly caused by the unfamiliarity of this rare entity and the confusing and self-contradictory features of the disease. While clinical features such as vomiting suggest small bowel obstruction, the radiographic findings are that of colonic distension, which is uncommon in small bowel obstruction.^3^,^4^,^12^ Radiographically, ISK is often mistaken for simple sigmoid volvulus. However, unlike sigmoid volvulus, attempts to deflate the distended colon using a sigmoidoscope or a flatus tube, often fails in ISK. This is because the ileum tightly envelops the base of sigmoid colon, defying any such attempt. These three features of the clinical picture of small bowel obstruction, radiographic evidence of predominately large bowel obstruction, and inability to insert a sigmoidoscope could possibly form a useful diagnostic triad.^12^

The radiographic findings of ISK, which include a double loop of dilated sigmoid shadow and multiple air fluid levels in the small intestine, are sporadically described and are difficult to identify as such because of unfamiliarity.^2^,^3^,^4^,^8^,^12^ Nonetheless, it is important that they should at least raise the suspicion of ISK. The findings in a CT scan suggestive of ISK include the whirl sign created by the twisted intestine and sigmoid mesocolon in ileosigmoid knot, medial deviation of the cecum, and descending colon.^11^,^13^,^14^ In addition, others have noted radial distribution of the intestine and mesenteric vasculature and consider it to be helpful diagnostic information.^11^ However, CT examination may not be possible in all patients as some could be in poor condition or because of nonavailability. Whenever possible, a CT examination could be useful in making a diagnosis in these otherwise difficult patients.^11^,^13^,^14^

The initial management involves aggressive resuscitation with fluid and electrolytes with the help of central venous pressure monitoring, if required, and the correction of acid-base imbalance if any. After hemodynamic stabilization, laparotomy should not be delayed. Appropriate antibiotic therapy is commenced early and continued after the operation. The usual antibiotic combination includes cephalosporins, aminoglycosides, and metranidazole.^5^,^7^,^12^

The anatomical and pathological changes dictate the operative procedure.^2^–^13^ In 73.5% to 79.4% of the cases, gangrenous bowel was encountered, whereas in 20.6% to 26.5% both small and large bowel were assessed to be viable in surgery.^2^,^4^ In 52.9% to 60.3% cases, both the small intestine and sigmoid colon were gangrenous.^2^,^4^,^8^,^9^ Paradoxically, the incidence of bowel gangrene was 90.9% in those who presented within 24 hours of their symptoms. Among those who presented after 24 hours after their initial symptoms, bowel gangrene was seen in 57% (chi-squared 9.94, *P* <.01).^2^,^4^

Various surgical procedures have been conducted in these patients (Table 3). If both loops are viable the knot may be undone by sigmoid enterotomy and traction of the sigmoid loop. This procedure may also be selected when the sigmoid colon alone is viable. When the ileum and the sigmoid colon are gangrenous, it can be difficult to untie the knot, and rupture of the gangrenous loop could lead to spillage of toxic bowel contents.^3^–^6^ Therefore, intestinal clamps should be applied before dissection or resection of the knot followed by resection of both the loops.

**Table 3:** Surgical procedures and outcome.

								Outcome	
Author	No.	IR+PA SD	IR+PA MS	IR+EI SR+PA	ID SR+PA	IR+PA SR+PA	IR+PA SR+HR/Col	Good	Death
Bawa, 2008	1					1		1	
Atamanalp, 2006	9	1				1	7	8	1 (11.1%)
Berrebi, 2006	1						1	1	
Hirano, 2005	1					1		1	
Atamanalp, 2004	63	10	2	1		6	38	53	10 (15.9%)
Hashimato, 2004	2					2		2	
Tamura, 2004	1					1		1	
Raveenthiran, 2001	7				4	3		7	
Akgun, 1997	16	4	3	3	3	3		13	3 (18.7%)
Alver, 1993	68	10			18	26	33	47	21 (30.8%)
Miller, 1992	1					1		1	
Puthu, 1991	7					4	2	5	2 (28.7%) (1 on table)
Kakar, 1981	11	2	2				7	8	3 (27.2%)
Shephard, 1967	92							48	44 (47.8%)

IR-ileal resection; PA-primary anastomosis; SD-sigmoid colon detorsion; MS-mesosigmidostomy; EI-end ileostomy; HR-Hartman's procedure

Primary anastomosis of the small bowel is preferable, but if the terminal ileum is gangrenous to within 10 cms of the ileocecal valve, an end-to-end anastomosis should not be attempted.^2^–^4^ The distal stump should be closed and end-to-side ileocaecostomy should be performed. Resection of the sigmoid colon is often advised in all instances even when viable. Recurrent volvulus or repeat knotting due to redundancy of the loop may cause gangrene after surgery. In the past, a Hartmann operation or a covering colostomy was advocated to avoid the risk of fecal leak from colonic anastomosis. However, recent data suggest that primary colonic anastomosis may be undertaken safely when the history is short and the remaining bowel is clean, well vascularized and undistended.^11^–^15^ Intraoperative colonic irrigation, followed by resection and primary anastomosis, is another alternative. Avoiding a colostomy is always welcome as it reduces the morbidity and cost of health care.^12^ In patients without sigmoid gangrene, mesosigmoidostomy is practiced by some to prevent recurrent ISK.^5^,^6^,^8^,^9^

The reported mortality from ISK varies from 0% to 48% (mean, 35.5%) (Table 3). The mortality figures are generally related to the duration of symptoms, the presence or absence of gangrene and the general status of the patient, including the presence of septicemic shock.^2^–^9^ However, some of the reports suggest that those patients who undergo surgery within 24 hours after the onset of symptoms have a significantly higher incidence of mortality than those whose symptoms exceed 24 hours.^2^–^4^ In spite of early judgment for prompt surgical intervention, the higher rates of both gangrene and mortality seem paradoxical and presumably reflect the fulminating clinical deterioration of patients due to early and extensive infarction of the bowel involved in a tight knot. However, others have found no correlation between the duration of symptoms and incidence of gangrene and consider the incidence and onset gangrene to be related more to the geometric degree of rotation than the duration of symptoms.^12^ The literature review reveals that there has been a constant decline in the mortality with few reported cases of adverse outcome in the last ten years (Table 3).

In summary, ISK is a rare cause of intestinal obstruction. Unfamiliarity and diagnostic difficulties have contributed to the high morbidity and mortality of this condition in the past. Better understanding of the problem, and increased probabilities of preoperative diagnosis with aid of CT scanning, have facilitated early diagnosis and intervention. Aggressive fluid resuscitation, preoperative antibiotics, prompt laparotomy, and effective surgery, including resection and primary anastomosis or mesosigmoidoplasty of viable sigmoid colon and better perioperative care of the shocked patient have optimized the survival of these patients.
